# EuroCrops: The Largest Harmonized Open Crop Dataset Across the European Union

**DOI:** 10.1038/s41597-023-02517-0

**Published:** 2023-09-11

**Authors:** Maja Schneider, Tobias Schelte, Felix Schmitz, Marco Körner

**Affiliations:** Technical University of Munich (TUM), TUM School of Engineering and Design, Munich, 80333 Germany

**Keywords:** Biodiversity, Plant development, Agriculture, Research data

## Abstract

EuroCrops contains geo-referenced polygons of agricultural croplands from 16 countries of the *European Union (EU)* as well as information on the respective crop species grown there. These semantic annotations are derived from self-declarations by farmers receiving subsidies under the *common agricultural policy (CAP)* of the *European Commission (EC)*. Over the last 1.5 years, the individual national crop datasets have been manually collected, the crop classes have been translated into the English language and transferred into the newly developed *hierarchical crop and agriculture taxonomy (HCAT)*. EuroCrops is publicly available under continuous improvement through an active user community.

## Background & Summary

As the world’s population continues to grow and global climate change becomes increasingly apparent, enhancing the efficiency and resilience of agriculture at both the local and global level is a crucial challenge for humanity’s future. Recent developments in satellite-based *Earth observation (EO)* have provided us with the ability to observe and analyse the processes occurring on the Earth’s surface in near real-time. By leveraging machine learning and artificial intelligence, we can extract valuable insights from these enormous volumes of high-quality and information-rich data, which can inform the development of functional process models for the monitoring of agricultural crops and the design of future applications. For example, the activity of these vegetation stands could be monitored and deviations from the expected progression, and thus the expected crop yields, could be detected. Based on this information, farmers would be able to initiate countermeasures at an early stage. This would make a decisive contribution to food security, representing one of the central *sustainability development goals (SDGs)* stated by the *United Nations (UN)*. However, these possibilities are massively limited by the insufficient availability of qualitative reference data, which are necessary for the creation of functional process models on the basis of such Earth observation data.

The EuroCrops project aims to show how this gap can be filled by compiling administrative data assessed in the context of agricultural subsidy control in the *European Union (EU)* area. Therefore, publicly available *Land Parcel Identification System (LPIS)* data, the essential part of the spatial information used to support *Integrated Administration and Control System (IACS)* applications under the *common agricultural policy (CAP)*, is individually collected from the member states. It contains georeferenced blocks of agricultural parcels that have been identified and are eligible for EU aid application. This data usually shows the main crop for a certain year as the subsidy is granted with respect to that.

A first pilot project^1^ exemplified the process compiling a dataset from that type of data. For this purpose, we collected geo-referenced crop datasets from three countries within Europe, harmonised the data by translating the crop names and developed an hierarchical structure to order the occurring crops. Finally, the crop labels were paired with the corresponding Sentinel-2 EO data and we released the TinyEuroCrops^2^ dataset publicly via the repository of the Technical University of Munich. Despite faced with some challenges, we soon realised that the dataset gained its popularity not due to the satellite data, but due to the fact that we also published the geo-referenced field polygon vector data together with the harmonised information of which crop species were cultivated there for a certain year. Having this data prepared in one reconciled format, language, and centrally available across borders and not just on a national level sparked the discussions about its broad applicability in various domains. The fact that this data has been prepared in a joint standardised format and language and that it is centrally available across borders and not only at national level has triggered discussion about its broad applicability in various areas. The research questions related to the analysis of agricultural diversity and food security in Europe were one of the reasons for the popularity of the dataset, leading to the motivation to extend them spatially and later also temporally.

**Fig. 1:**
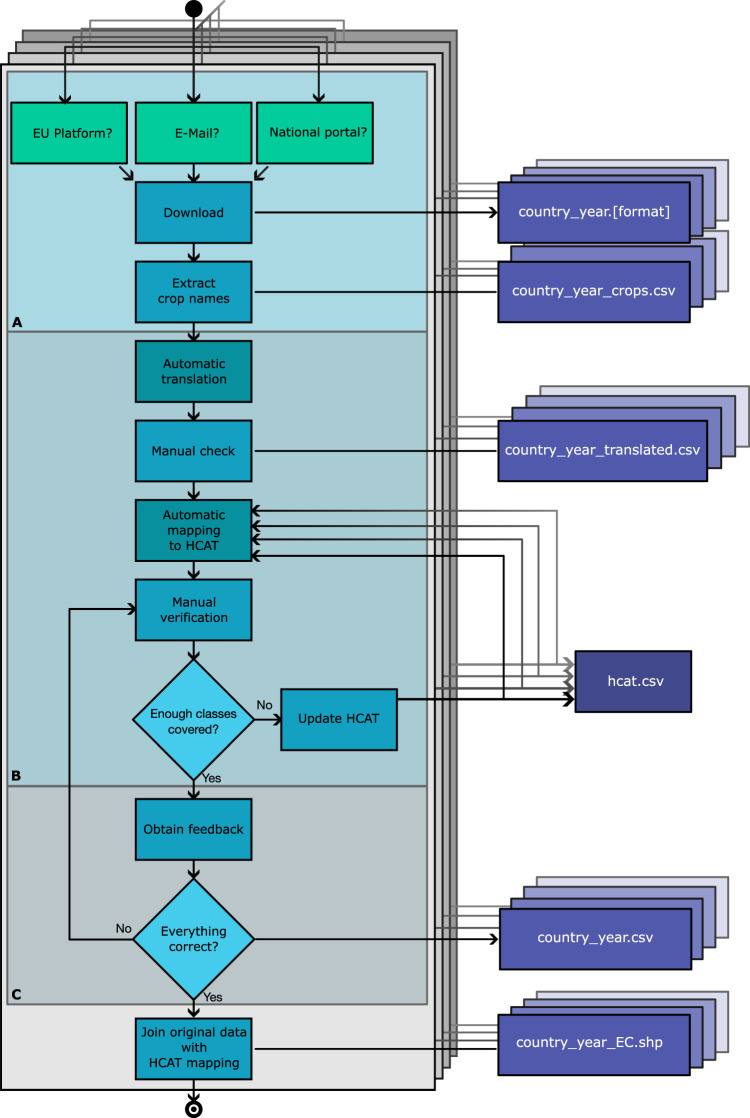
The process of constructing the EuroCrops dataset. Each layer represents the process for one country with the three stages of development: **A. Data Collection,** **B. Harmonisation,** **C. Validation and Feedback**. Each stage has one or more outputs, indicated in purple, and with country and year being replaced accordingly. Only the hcat.csv exists once across all country-specific processes and gets gradually updated in each harmonisation step. While automatic sections exist, a manual check is required each time, making the progress in total heavily dependent on work that has to be done by hand.

In this article, we present and describe the first spatially extended EuroCrops vector dataset. For this release, we manually collected the raw crop declaration data from 16 EU countries, which was made available and distributed across multiple platforms and servers. In light of previous studies, e.g., BreizhCrops^3^, ZueriCrop^4^, and CropHarvest^5^, the key objectives of EuroCrops lie in the extension of both the variability of crop species classes to be represented and the geographical scale of the considered regions. After translating the textual declarations data, we developed a new version of our *hierarchical crop and agriculture taxonomy (HCAT)*^1^ in order to organize all crops that are cultivated within the EU into a common hierarchical representation scheme. The process of this development is visualised in Fig. 1 and will be further explained in the methods section.

By being able to analyse agricultural data at this expanded spatial scale, which extends from Sweden to Portugal, we hope to enable researchers to carry out their work across borders and gain new insights. EuroCrops is ongoing and will be extended on a regular basis. We are putting effort into increasing the spatial and temporal coverage of the dataset as well as the preparation of analysis-ready data by combining it with Sentinel-2 data. Updates will be provided on Zenodo and the GitHub repository associated with the project.

## Methods

In order to compile the presented dataset, several iterative steps had to be performed, which can roughly be grouped into *data collection*, *harmonisation* and *validation*, denoted as **A,** **B** and **C** in Fig. 1 and will be further described in the next subsections. Data obtained from each member state of the EU has to undergo the entire procedure, sometimes even multiple times, as indicated by the stacked layers in Fig. 1 and arrows going from each countries Update HCAT process back to the beginning and the Automatic mapping to HCAT for the individual dataset. This recurring loop is the main reason for the exponentially increasing amount of manual work that was necessary for the creation of the dataset and required careful deliberation on the right moment for cutting the development of HCAT.

### A. Data collection

As EuroCrops consists of multiple smaller datasets, the data collection itself plays an integral role. This paper will focus on the practical part of that process, whereas an in-depth analysis of the challenges of creating a transnational dataset is described in more detail by Schneider *et al*.^6^.

**Fig. 2:**
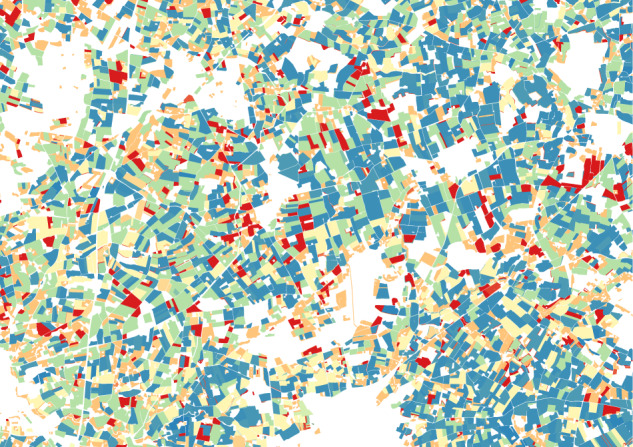
Exemplified raw input data with the corresponding attribute table shown in Table 5. This selections shows parts of the North Rhine-Westphalian (Germany) dataset^14^ with each crop class being coloured differently. The data consists of geo-referenced polygons which indicate the field borders and hold information about the grown crop for a certain year.

**Fig. 3:**
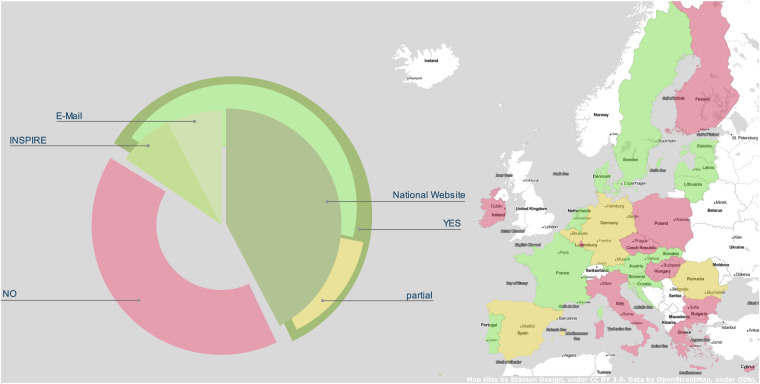
This diagram shows the data availability, coverage and access across the EU. The map on the right indicates whether data from a certain country is part of EuroCrops. Green indicates a full country participation, yellow are the member states with only partial coverage and data from red countries was not available at the time of the development of the dataset. Finland for instance released the data later and is still covered in the section “Data Records”, but is not part of EuroCrops. On the left, the pie chart breaks down several analyses: Generally, red and green shades tell what fraction of data in the EU is available, while indicating with bright green and yellow full or partial coverage, as already on the map. The green pie segments on top visualise where the data was originally obtained from. This shows, similar to Table 1 that the majority of the data originates from national websites, including geoportals.

Generally, we identified four ways of data acquisition: Firstly, many countries publish national crop data on the webpage of the respective ministry or agency responsible for agricultural, food or rural topics. Some countries instead offer a national geoportal, distributing different kinds of geodata specifically or, as another mean of distribution, publishing geodata on an international level, e.g. via INSPIRE^7^ or data.europe.eu^8^. Lastly, if the data is not openly distributed on a webpage or geoportal, we reached out personally to ministries or agencies and asked for the data directly. Most of the national datasets used in the EuroCrops project were collected from national ministry webpages or geoportals as listed in Tables 1, 2 respectively, mostly made available as *ESRI shapefiles*, GeoJSON, or GeoPackage (GPKG). Nonetheless, some data can only be accessed via a *web feature service (WFS)* implemented in a *geographic information system (GIS)*, allowing the user to display the desired data and save it in a chosen file format. The other means of data access are shown in Tables 3, 4. Figure 3 puts all this information into context, gives an overview of the available datasets, and indicates from where the data originates. Countries marked yellow in Fig. 3 indicate only partial availability of crop data for the respective country. In order to give a better understanding of the original raw datasets we got from the countries, we visualised a small fraction of the data from North-Rhine Westphalia (Germany) in Fig. 2 with coloured geo-referenced agricultural parcel polygons. Table 5 gives an impression of how the corresponding original raw attribute table looks like. Each row entry describes the crop species that has been cultivated on the associated parcel.

**Table 1:** National (ministry) website: The majority of the EuroCrops data sources were websites, usually hosted by the respective ministry or agency.

Country (state)	National agency	URL	Format
Belgium (Flanders)	Department of Agriculture and Fisheries (Departement Landbouw & Visserij)	see Departement Landbouw en Visserij^11^	shapefile, GPKG
Croatia	Agency for Payments in Agriculture, Fisheries and Rural Development	https://www.apprrr.hr/prostorni-podaci-servisi/	GPKG
Denmark	Danish Agricultural Agency	https://landbrugsgeodata.fvm.dk/	shapefile
Finland	Finish Food Authority	https://kartta.paikkatietoikkuna.fi/	WFS
Latvia	Rural Support Service Republic of Latvia (Lauku atbalsta dienests)	https://www.lad.gov.lv/lv/lauku-registra-dati	WFS
Netherlands	Ministry of economic affairs and climate (Ministerie van Economische Zaken en Klimaat) via: PDOK platform (Publieke dienstverlening op de kaart)	https://www.pdok.nl/introductie/-/article/basisregistratie-gewaspercelen-brp-	WFS
Portugal	Portuguese Finance Institute of Agriculture and Fisheries (Instituto de Financiamento da Agricultura e Pescas)	https://www.ifap.pt/isip/ows/	WFS
Slovenia	Ministry of Agriculture, Forestry and Food (Ministrstvo za Kmetijstvo, Gozdarstvo in Prehrano)	https://rkg.gov.si/vstop/	shapefile

These websites are usually in the national language, without any English translation which makes the discoverability and accessibility of the data laborious for international researchers.

**Table 2:** National geoportal: Some countries or regions actively participate in Europe’s open data initiative and publish their crop data on a national geoportal.

Country (state)	Portal	URL	Format
Austria	data.gv.at	see Agrarmarkt Austria^10^	GPKG
Belgium (Wallonia)	Géoportail de la Wallonie	https://geoportail.wallonie.be/catalogue-donnees-et-services	shapefile
France	data.gouv.fr	see Agence de services et de paiement (ASP)^13^	shapefile
Germany (North Rhine-Westphalia)	OpenGeodata.NRW	see Landwirtschaftskammer NRW^14^	shapefile
Germany (Lower Saxony)	Landentwicklung und Agrarförderung Niedersachsen-Portal	https://sla.niedersachsen.de/landentwicklung/LEA/	shapefile
Lithuania	geoportal.lt	see Nacionalinė mokėjimo agentūra prie Žemės ūkio ministerijos^16^	shapefile
Spain	“Sicpac” portal for each Autonomous Community, e.g. Navarra	https://filescartografia.navarra.es/2_CARTOGRAFIA_TEMATICA/2_6_SIGPAC/	

The goal of these portals is to make data available to the public sector and lower the entry barrier to letting citizens actively participate.

**Table 3:** Direct contact: Data from Slovakia and Sweden was directly sent to the project members by a contact person in the respective country.

Country	Authority	Format
Slovakia	National Agricultural and Food Centre	shapefile
Sweden	The Swedish Board of Agriculture	shapefile

**Table 4:** International Platform This table lists all the countries which data was acquired through an international platform.

Country	Portal	Format
[Austria	data.europa.eu^9^	GPKG]
Estonia	INSPIRE Geoportal^12^	WFS
Romania	INSPIRE Geoportal^17^	shapefile

The data for Austria would be available via “data.europa.eu”, but for EuroCrops we downloaded it directly from the national website.

**Table 5:** After downloading the respective national raw datasets, we first examined the attribute tables and extracted the values of the columns representing the cultivated crops.

ID	FLIK	AREA_HA	CODE	CODE_TXT	USE_CODE	USE_TXT	WJ	DAT_BEARB
4597509	DENWLI0543050566	2.1808	411	Silomais (als Hauptfutter)	AF	Ackerfutter	2021	2021/03/01
4597510	DENWLI0543051616	1.5319	459	Grünland (Dauergrünland)	GL	Dauergrünland	2021	2021/03/01
4597641	DENWLI0542022516	1.0293	480	Streuobst mit DGL-Nutzung	GL	Dauergrünland	2021	2021/03/02
4597657	DENWLI0541093620	2.4966	459	Grünland (Dauergrünland)	GL	Dauergrünland	2021	2021/03/02
4597810	DENWLI0540163053	1.162	121	Winterroggen	GT	Getreide	2021	2021/03/04

In the given example, showing raw data from North Rhine-Westphalia (Germany)^14^, multiple columns representing the cultivated crops can be determined. Thus a selection had to be made. In this case, values from the “CODE_TXT” column were translated and matched with the occurring classes in HCAT. Each time we discovered a class that was not represented in the taxonomy yet, we included it and started the harmonisation process again. The corresponding vector data to this file is illustrated in Fig. 2 and the same attribute table enriched with the EuroCrops columns is shown in Table 6.

From all red coloured countries in Fig. 3 it was not possible to obtain publicly available data. There are several reasons for that, such as *General Data Protection Regulation (GDPR)* issues, a missing incentive to publish the datasets and sometimes no response over years from the responsible authorities. However, as these types of data collections have recently been declared high-value datasets by the *European Commission (EC)* (https://digital-strategy.ec.europa.eu/en/news/commission-defines-high-value-datasets-be-made-available-re-use), we expect a change towards a more open publishing culture in the future.

In the following paragraphs, all individual sources for the available datasets are presented. For each contributing country the data source, available years, coverage, licence, and format are described and referenced. By doing so, we aspire to give the research community a tool to discover and access raw data faster and more reliably.

#### Austria

The dataset for Austria comprises a vast range of years, spanning from 2015 to 2021. Moreover, the whole territory of the country is covered without any regional omissions. Crop classes are defined very detailed with an approximate number of 200 classes. The files were made available in GPKG format via two platforms, the European “data.europa.eu”^9^ and “data.gv.at”^10^, a platform that distributes data of the public sector in Austria for further analysis and development. However, both platforms receive the datasets from “Agrarmarkt Austria”, which is a public geodata office. As such, data is published free of charge under the Creative Commons Licence CC-BY-AT 4.0. In the course of the EuroCrops project, the dataset of 2021 was harmonised for Austria.

#### Belgium

Due to the federal structure of Belgium, the data is split into two sets covering the regions of Flanders and Wallonia. Not only is the data published via different platforms, its structure also differs heavily between the two regions.

The data for Flanders^11^ is published by the Department of Agriculture and Fishery on its website as shapefiles. Is is anonymous and can be used freely. Additionally, a word document explaining the current state of the data as well as the abbreviations that occur in the attribute table of the shapefiles is available in Flemish language. The crop classes are differentiated very precisely with an approximate number of 275 classes. Datasets are available for the years 2019, 2020 and 2021.

The datasets for Wallonia are published by the Geoportal of Wallonia (https://geoportail.wallonie.be/catalogue-donnees-et-services) as shapefiles, but a registration is required. With an approximate number of 150 classes the crop classification of Wallonia is still quite precise, even though the Flemish data is more detailed. On the other hand, a wider time period is captured by the Wallonian datasets, covering all years since 2015.

So far only the Flemish data for the year 2021 got harmonised in the course of the EuroCrops-Project.

#### Croatia

The Croatian data is distributed in GPKG format via a platform managed by the Agency for Payments in Agriculture, Fisheries and Rural Development (https://www.apprrr.hr/prostorni-podaci-servisi/), where an abundant sequence of years is available ranging from 2011 to 2021. Due to translation difficulties, we obtained the data directly from the Paying Agency with the rights to include it in EuroCrops. While all regions of the country are covered by the dataset, its differentiation between 14 crop classes turns out to be rather coarse. For EuroCrops, the data of 2020 was harmonised.

#### Denmark

The dataset of Denmark comprises of only the mainland. The Faroe Islands and Greenland are not included. However, with an approximate number of 300 classes, the Danish crop taxonomy is very detailed. Datasets are available since the year 2017. The data is available as shapefiles provided by the Danish Agricultural Agency (https://landbrugsgeodata.fvm.dk/). All the data provided is considered open data, which means it can be openly used and distributed. The Danish data of 2019 was harmonised throughout the course of the EuroCrops Project.

#### Estonia

The Estonian dataset^12^ is made available under the “Autorile viitamine-Jagamine samadel tingimustel 3.0 Eesti” which corresponds to a CC-BY-SA licence. Thus, there are no limitations to public access. It can be acquired via the INSPIRE Geoportal as WFS. When accessing the data via a WFS URL in a GIS, the dataset can be transformed and saved as GeoJSON for example. It covers all of Estonia but only for the current year. Thus, data from 2021 was harmonised. However, the crop differentiation is very precise, leading to a high number of ca. 150 classes.

#### Finland

The Finnish dataset covers all provinces of the country. Data is available for the years 2020 and 2021. However, none of the years got harmonised yet, as Finland provided its datasets very late after the harmonisation process was already completed. The data differentiates between 200 classes roughly, which enables a very precise crop classification. The Finish Food Authority distributes the data via a WFS (https://kartta.paikkatietoikkuna.fi/) under the Creative Commons Licence BY 4.0. Consequently, the datasets were implemented into a GIS and saved as shapefile.

#### France

France publishes national geodata as open licence on the “data.gouv.fr” platform^13^ as GPKG- and shapefiles. While the central point of distribution makes it easy to discover and access the data, the fact that each region has its own sub-dataset makes the platform barely usable for someone who needs the entirety of the French data. Luckily, there is a second (unofficial) server (data.cquest.org/registre_parcellaire_graphique/2018/data.cquest.org/registre_parcellaire_graphique/2018/) that hosts a combination of all these national datasets in shapefile format. Additionally, an excel sheet is available, containing the descriptions of all crop abbreviations used in the datasets. The class differentiation is moderate. Approximately 70 crop classes are distinguished. In the course of the project, datasets were downloaded for the years spanning from 2016 to 2019, of which the file for 2018 was harmonised. The data covers not only the French mainland but also overseas territories.

#### Germany

Due to the federal structure of Germany datasets are not published on a national level, but by each federal state (“Bundesland”) individually. Two datasets were acquired: One covers Lower Saxony (https://sla.niedersachsen.de/landentwicklung/LEA/) and another one North Rhine-Westphalia^14^. Both datasets depict the crop situation of 2021 and have a very high class precision, distinguishing between ca. 240 crop classes. Both files are distributed as shapefiles, one on the online platform for Rural Development and Agricultural Promotion of Lower Saxony, the other one on the geoportal of North Rhine-Westphalia. Both datasets are published under “data licence Germany - attribution - Version 2.0”. For the sake of completeness, it is worth noting that Brandenburg also published its data^15^, but has not been included into EuroCrops yet.

#### Latvia

The Rural Support Service of Latvia (https://www.lad.gov.lv/lv/lauku-registra-dati) provides a WFS, which can be used to implement and convert the Latvian files to GeoJSON or shapefile in a GIS. The data is open so there are no publishing restrictions. The files cover the whole territory of the country and are available for 2021 and 2022. The file for 2021 got harmonised in the course of the EuroCrops Project. The class precision is very high, differentiating between 150 crop types approximately.

#### Lithuania

The crop parcels of Lithuania^16^ are available as shapefiles for the year 2021 covering the whole territory of the country. Consequently, data got harmonised for the aforementioned year. The file differentiates between 24 crop classes only. However, the chosen classes are precise. Datasets of a similar low number of classes normally assign very general crop terms to the classes (i.e. vineyard, citrus fruits, grassland). In the case of Lithuania, the crop types assigned to the classes are very specific. Thus, the class precision can be defined as medium, despite its low number of actual classes. The data is published via Geoportal.lt under their own copyright, but it requires registration.

#### Netherlands

The Dutch Ministry of Economic Affairs and Climate distributes datasets via a WFS on the platform PDOK (https://www.pdok.nl/introductie/-/article/basisregistratie-gewaspercelen-brp-). The files comprise only the mainland of the Netherlands; overseas territories are not included. The class precision is very high, encompassing around 320 different plant categories. So far data is only available for the years 2020 and 2021, of which the file for 2020 was harmonised for EuroCrops. The datasets fall under the CC0 1.0 licence category which does not impose any limitations to public access.

#### Portugal

The Portuguese datasets are available since 2017, with the file for 2021 harmonised. Data since 2020 covers the complete national territory of Portugal. Contrarily, the files for 2017, 2018 and 2019 are split up into regional territories, which had to be merged in a first step. Moreover, some of the Portuguese regions are missing whereas the national datasets provide a complete and uniform depiction of Portuguese crop cover. Furthermore, the class precision differs between the regional and the national datasets. The crop differentiation is moderate for the regional sets with ca. 50 to 150 classes, whereas it is more precise for the national datasets with more than 200 classes. The files can be accessed via a WFS (https://www.ifap.pt/isip/ows/) provided by the Portuguese Finance Institute of Agriculture and Fisheries and is usable without legal restrictions.

#### Romania

Romania officially does not yet publish crop data but is, according to the *Agenţia de Plăţi şi Intervenţie pentru Agricultură*, actively working towards it. We therefore decided to add an unlicensed, coarse and only regional land cover dataset^17^ into EuroCrops in order to give an incentive and an idea of how Romanian data would be integrated in the future.

#### Slovenia

The Slovenian dataset covers the territory of the whole country and the years 2019, 2020 and 2021. The file for 2021 got harmonised. The class precision is high, with approximately 150 different crop classes. The files are distributed as shapefiles at the website of the Ministry of Agriculture, Forestry and Food (https://rkg.gov.si/vstop/). Additionally, two text files are published which describe the crop codes assigned to the plants with one file being in Slovenian language, the other one in English. All data is made publicly available without use restrictions, however, citing the source is required.

#### Slovakia

Slovakian data is available for the years 2020, 2021 and 2022. The datasets cover all regions of the country. The file depicting the crop situation in 2021 was harmonised. The class precision is very high, differentiating between roughly 170 crop types. The data was sent directly to the project members via e-mail by the Slovakian Agricultural Paying Agency with the permission to include it into EuroCrops.

#### Spain

Spain distributes data under the licence CC BY 4.0 separately for each of its autonomous communities where each one has their own website. The crop parcel data can be downloaded there as a shapefile in most cases. The Navarra dataset (https://filescartografia.navarra.es/2_CARTOGRAFIA_TEMATICA/2_6_SIGPAC/) for 2021 got harmonised. However, the data is very coarse, differentiating between 21 classes only.

#### Sweden

GeoJSON files covering the crop parcels of all of Sweden for the years 2020 and 2021 were sent by a contact person at the Swedish Board of Agriculture to the project members by email. The files have a medium class precision distinguishing between ca. 80 classes, are published under the CC BY 4.0 licence and data depicting the crop situation in 2021 got harmonised.

### B. Harmonising country-specific crop classes

After collecting the data and extracting the set of country-specific crop classes from the attribute tables, we initiated the harmonisation process. This step is necessary because the crop names from each country usually come in the national language of the member states and without standardised codes, as shown in Table 2.

Instead of working with the entire attribute table, we worked with a table showing the name and code of a certain crop class per row together with its absolute and relative occurrence in the dataset. In Fig. 1, that file, preserving original crop class name and code, is denoted as country_year_crops.csv.

Following this, the automatic translation starts the process of harmonising the given original crop class names into the HCAT taxonomy. Therefore, multiple steps had to be performed: The file country_year_translated.csv arises from the translation of the crop classes into English. Despite the access to modern translation programmes, we were not able to automate this part end-to-end, as country-specific agricultural terms seem to cause mistranslations across all common translators. By correcting the translations manually, we hope to bridge that gap and make the dataset as reliable as possible. Similarly, mapping the translations to HCAT was only possible to perform automatically to a certain degree and required manual checks (see Fig. 1) as well. This was again caused by the diversity of the crop classes declared by the member states.

Within this process of manual translation and matching, we were able to catch most of the missing classes in our growing taxonomy. The iterative updates of HCAT helped in the detailed classification of delivered datafiles by the countries, but also shed light on relevant and focus areas within the taxonomy. To the matching HCAT name, we also added the corresponding HCAT code, which embeds the hierarchy of the taxonomy. This way, we enriched the country-specific original crop name and code with our HCAT name and code and the absolute and relative occurrence in a country.

Hence, we are able to visualise the number of instances of certain crop classes and compare the occurrences with those from other countries for general diversity analysis and taxonomy class updates. The preliminary file is stored in a country_year.csv after positive assessment during working step **C**.

### C. Community work: content validation and feedback incorporation

The largest expertise on country-specific crop classes still lies with the respective countries, driving to the decision to keep them onboard during the validation phase of the project. Therefore, we asked all countries during the end phase of our pipeline if our translations and mappings seemed reasonable. Out of 16 countries, we received feedback from seven who double-checked and reviewed our work. While this increased the quality of the dataset, it also started another loop in the harmonisation block, which is visualised in Fig. 1 as the arrow going from Everything correct? to Manual verification. Eventually, we uploaded the first version of the dataset on our university-owned data-sharing platform and set up a GitHub repository (https://github.com/maja601/EuroCrops) for the community to have a first look. This resulted into several opened issues and pull requests where improvements to the mappings were suggested. Each time we were content with a version of the mapping, we manually joined the original dataset with our mapping and saved it as a shapefile. This lead to one shapefile for each country and five successive versions of the dataset incorporating the proposed changes from GitHub. One exemplary attribute table of such a shapefile is shown in Table 5. All of the versions were individually uploaded to Zenodo^18^, which now officially tracks the versions with a *Digital Object Identifier (DOI)*.

## Data Records

The EuroCrops dataset is currently published as individual country- or region-covering shapefiles and hosted on Zenodo^18^ with dynamic updates available on GitHub (https://github.com/maja601/EuroCrops). Therefore, all individual file types that are required (.shp,.shx,.dbf) and optional (.cpg,.prj) to form a shapefile are zip compressed as one sub-dataset directory and can be downloaded individually. This way, researchers that are only interested in a specific area can make use of a selection of EuroCrops without having to download everything. The naming convection for the individual files is the country name in ISO-3166 Alpha-2 format with an optional regional identifier and the year for which the data has been harmonised. The attribute tables of the original shapefiles which have been introduced in Section **A. Data Collection** are not altered throughout the process, but amended by the three columns EC_trans_n, EC_hcat_n, EC_hcat_c, representing the translated crop name, the HCAT name and the HCAT code respectively as shown in Table 6.

**Table 6:** This table shows an example of a final EuroCrops data attribute table.

ID	…	CODE_TXT	USE_CODE	USE_TXT	WJ	DAT_BEARB	EC_trans_n	EC_hcat_n	EC_hcat_c
4597509	…	Silomais (als Hauptfutter)	AF	Ackerfutter	2021	2021/03/01	Silage maize (as staple feed)	green_silo_maize	3301090400
4597510	…	Grünland (Dauergrünland)	GL	Dauergrünland	2021	2021/03/01	Grassland (permanent grassland)	pasture_meadow_grassland_grass	3302000000
4597641	…	Streuobst mit DGL-Nutzung	GL	Dauergrünland	2021	2021/03/02	Orchards with Permanent grassland use	orchards_fruits	3303010000
4597657	…	Grünland (Dauergrünland)	GL	Dauergrünland	2021	2021/03/02	Grassland (permanent grassland)	pasture_meadow_grassland_grass	3302000000
4597810	…	Winterroggen	GT	Getreide	2021	2021/03/04	Winter rye	winter_rye	3301010301

While the original columns as shown in Table 5 remains the same (“FLIK”, “AREA_HA” and “CODE” have been abbreviated in this print), three additional attributes were added: Firstly, “EC_trans_n” is the direct translation of the crop name in its original language. Then, correspondingly, “EC_hcat_n” is the matched name of that particular crop in *HCAT*. These names are all lowercase and with underscores to make them easier to process automatically. Lastly, the column “EC_hcat_c” shows the HCAT code that puts the HCAT names into a hierarchical structure. A more detailed explanation of HCAT is presented in the publication by Schneider *et al*.^1^.

## Technical Validation

Regarding the correctness of the underlying original data, it is important to stress that self-declarations build the basis of the input. From official site, the *in-situ* controls act as a validation instance to these declarations, but these are just sparse samples and would never be able to cover the entire area. One approach to actually validate the original data on a bigger scale was introduced by Gounari *et al*.^19^, but this would exceed the project tasks. On our side, we concentrated on a valid harmonisation of the entire dataset. The validation of the content itself was already discussed in the methods section: We incorporated the knowledge from the respective authorities and updated the mappings based on feedback from the community.

## Usage Notes

The data is currently published as one shapefile per country on Zenodo^18^ which can, for instance, be opened with QGIS^20^. On Zenodo, EuroCrops version 6 is associated with this peer-reviewed article. The corresponding, dynamically updated mapping files on GitHub (https://github.com/maja601/EuroCrops/tree/main/csvs/country_mappings) are in CSV format with the structure found in Table 7. In order to use data from a year that has not been harmonised within EuroCrops, it is possible to join the mapping file of a country with the raw vector data file which can be found on the provided national platforms. By using the correct column in the original dataset, which is indicated in the wiki (https://github.com/maja601/EuroCrops/wiki) entry under “Attribute Table” for each country, also other datasets can be harmonised. This might lead to some missing crop types, as our taxonomy only holds the crop classes occluding in the stated sub-datasets, but we assume that the majority of the crops should be covered.

**Table 7:** In our aforementioned GitHub repository, we publish for each country a so-called mapping file.

original_code	original_name	translated_name	HCAT2_name	HCAT2_code
459	Grünland (Dauergrünland)	Grassland (permanent grassland)	pasture_meadow_grassland_grass	3302000000
411	Silomais (als Hauptfutter)	Silage maize (as staple feed)	green_silo_maize	3301090400

This file contains the set of occurring crops in one original file, together with its translation and the corresponding HCAT name and code. Note, that even though it says HCAT2 in the column names, it is the same as the previously mentioned HCAT. As the initial, prototyped taxonomy is not used any more. The shown example is an extraction of the sub-dataset which is already presented in Fig. 2 and Tables 5, 6. The entire file is available on GitHub and could for example be used to translate and map a dataset from North Rhine-Westphalia (Germany)^14^ of another year than 2021.

## Data Availability

For this study, no custom code was generated.
